# Comparisons and Uncertainty in Fat and Adipose Tissue Estimation Techniques: The Northern Elephant Seal as a Case Study

**DOI:** 10.1371/journal.pone.0131877

**Published:** 2015-06-29

**Authors:** Lisa K. Schwarz, Stella Villegas-Amtmann, Roxanne S. Beltran, Daniel P. Costa, Chandra Goetsch, Luis Hückstädt, Jennifer L. Maresh, Sarah H. Peterson

**Affiliations:** 1 Institute of Marine Sciences, University of California Santa Cruz, Santa Cruz, California, United States of America; 2 Department of Ecology and Evolutionary Biology, University of California Santa Cruz, Santa Cruz, California, United States of America; 3 Department of Biological Sciences, University of Alaska, Anchorage, Alaska, United States of America; New York Institute of Technology College of Osteopathic Medicine, UNITED STATES

## Abstract

Fat mass and body condition are important metrics in bioenergetics and physiological studies. They can also link foraging success with demographic rates, making them key components of models that predict population-level outcomes of environmental change. Therefore, it is important to incorporate uncertainty in physiological indicators if results will lead to species management decisions. Maternal fat mass in elephant seals (*Mirounga* spp) can predict reproductive rate and pup survival, but no one has quantified or identified the sources of uncertainty for the two fat mass estimation techniques (labeled-water and truncated cones). The current cones method can provide estimates of proportion adipose tissue in adult females and proportion fat of juveniles in northern elephant seals (*M*. *angustirostris*) comparable to labeled-water methods, but it does not work for all cases or species. We reviewed components and assumptions of the technique via measurements of seven early-molt and seven late-molt adult females. We show that seals are elliptical on land, rather than the assumed circular shape, and skin may account for a high proportion of what is often defined as blubber. Also, blubber extends past the neck-to-pelvis region, and comparisons of new and old ultrasound instrumentation indicate previous measurements of sculp thickness may be biased low. Accounting for such differences, and incorporating new measurements of blubber density and proportion of fat in blubber, we propose a modified cones method that can isolate blubber from non-blubber adipose tissue and separate fat into skin, blubber, and core compartments. Lastly, we found that adipose tissue and fat estimates using tritiated water may be biased high during the early molt. Both the tritiated water and modified cones methods had high, but reducible, uncertainty. The improved cones method for estimating body condition allows for more accurate quantification of the various tissue masses and may also be transferrable to other species.

## Introduction

Fat mass, adipose tissue mass, and body condition (often described as the relationship between body mass and body size or fat mass and body mass) are fundamental metrics used to investigate animal foraging success in relation to environmental variability because they indicate a level of energy reserve. Those metrics are also used in physiological studies, and body composition affects energetic costs, including transport, thermoregulation, and reproduction [1–3]. With respect to conservation, physiological indicators such as stress hormones and energy reserves (fat mass) are also used to link disturbance of individuals with population demographic effects [4].

For example, elephant seals (*Mirounga* spp) undertake long, oceanic foraging migrations and return to land biannually for extended periods of time to breed and molt. Since seals fast during these haul-outs, stored energy plays a key role in important life history functions. In particular, the fat mass of female elephant seals is a critical link between foraging success and reproductive rate. In addition, pup weaning mass is a function of maternal condition and affects pup survival, with the highest survival rates for pups of intermediate weaning mass [5, 6]. Consequently, fat mass can be used as a metric that relates foraging success, vital rates, and population-level changes [4]

However, complex systems cannot be understood perfectly, and management decisions must be made despite such uncertainty [7, 8]. Ideally, uncertainty is quantified and incorporated into risk assessment models, so policy makers can make more informed decisions regarding species and ecosystem management [9]. Therefore, if physiological indicators are used in models to predict the population outcome of disturbance, it is important to quantify the uncertainty in those metrics whenever possible.

Different methods have the ability to measure different forms of energy reserve. Chemical extraction techniques measure crude fat, or fat, which is a measure of hydrophobic molecules in a tissue, including triglycerides, lipid in cell membranes, steroids, and pigments [10]. Adipose tissue is connective tissue consisting mostly of adipocyte cells whose primary function is to store lipid, but it also includes vascular tissue, immune cells, and preadipocytes [11]. The proportion of fat in adipose tissue can vary, and mammalian adipose tissue is mostly found in the abdominal cavity and between the skin and muscle layers [11, 12]. Total adipose tissue mass can be difficult to measure when it is infused with other tissue types. Blubber is a type of adipose tissue specific to marine mammals that plays an important role in thermoregulation, locomotion, and energy storage [13]. Blubber can have a distinct boundary between skin and muscle layers, making blubber mass easier to determine [13]. However, additional adipose tissue may be found in other parts of the animal and can vary by species [13].

Isotope dilution methods are considered the “gold standard” for estimating body composition and partitioning the relative components of fat versus lean mass [14]. The technique measures the animals' total body water (*TBW*) by dilution of a known amount of isotopically-labeled water (either tritium or deuterium). After equilibration, *TBW* is calculated from the amount of isotope administered relative to the specific activity of the labeled water in some body fluid, typically blood [15]. *TBW* is then converted in to either total fat mass or total adipose tissue mass using a defined relationship.

The relationship between body water and adipose tissue has been determined from direct measurements of the proportion of water in adipose and non-adipose tissue [16] (S1 File). The conversion from *TBW* to adipose tissue mass or fat mass is created using eviscerated carcasses [17], wherein water mass is usually estimated by desiccation of homogenized tissue from half the carcass (to measure total body water) or various tissue components, and fat is extracted chemically from similar homogenized tissues. Both relationships have been measured for a large number of species and are highly consistent across species for adult mammals [14, 18]; however, many studies rely on the relationship determined from guinea pig (*Cavia porcellus*) carcass data [19]. The method can underestimate adipose tissue or fat content when additional pools of water are unaccounted for, such as milk in the mammary glands or water in the gut [20]. The relationships have been defined as linear functions that potentially allow for negative values of fat or adipose tissue, and uncertainty in the relationships has never been quantified.

Once the relationships between *TBW* and fat and adipose tissue mass have been established, isotope dilution provides a non-lethal method for estimating the fat or adipose tissue content of an animal. This method is often logistically difficult and time consuming, particularly in the field. As a result, another method was developed to estimate fat or adipose tissue mass in marine mammals by coupling morphometric data with measurements of blubber thickness obtained from ultrasound imaging, referred to as the truncated cones technique [21–23]. In seals, the outer volume of the cones (from the surface of the animal through the blubber layer) has been termed the blubber layer, although the measured tissue is the sculp (blubber plus skin) [24]. The volume of that region is then converted to blubber mass by multiplying by the density of that tissue [23, 24].

Gales and Burton [23] introduced the truncated cones technique to estimate total blubber mass in southern elephant seals (*M*. *leonina*). They first showed that dorsal measurements of blubber depth (defined as the distance between the surface of the skin and the muscle fascia) from two ultrasound instruments coincided well with each other on 16 domestic pigs (*Sus scrofa domesticus*). When compared with post-mortality endoscopic measures, the ultrasound measurements were lower, leading the authors to suggest fat slumping, when gravity pulls fat tissue down and reduces dorsal measurements, may have played a role in the discrepancy. They also compared an ultrasound measurement at one location with a depth measurement from an incision on an immobilized male southern elephant seal. Morphometric measures of girth, length, and blubber depth at six locations along 27 animals were converted to blubber volume and blubber mass, assuming no blubber anterior to the ears or posterior to the pelvis. They did not account for fat slumping.

Slip et al. [22] showed fat slumping in male southern elephant seals increased blubber volume estimates by roughly 5%. They also estimated that skin accounted for 18 and 21% of sculp mass in two fresh male carcasses. Direct measures of sculp mass were 16 and 25% higher than estimates using truncated cones accounting for slumping. Skin mass was estimated as a function of total mass [25], and fat mass was calculated as the difference between sculp and skin mass. In that case, fat mass was 8 and 2% lower than the two truncated cones sculp mass estimates. For comparison, *TBW* from isotope dilution was measured and converted to adipose tissue mass using a relationship developed by Ortiz et al. [16]. Comparisons of truncated cones and isotope dilution indicated that roughly 15% of the animal’s fat tissue mass was found outside the blubber.

Worthy et al. [24] calculated blubber mass in adult female northern elephant seals (*M*. *angustirostris*) using the truncated cones method with measurements at four locations along the animal, assuming no blubber anterior to the neck or posterior to the pelvis. For comparison, *TBW* from isotope dilution was measured and converted to adipose tissue mass using a relationship developed by Pace and Rathbun [19] from guinea pig data (S1 File). Blubber mass estimates from truncated cones and adipose tissue mass estimates from isotope dilution yielded similar results, which lead Worthy et al. [24] to suggest that northern elephant seals lack fat stores outside the hypodermis.

Webb et al. [21] converted blubber mass calculated from truncated cones to blubber fat mass for juvenile northern elephant seals, assuming blubber is 90% fat by mass. While the fat mass estimates from this truncated cones method were based on an increased number of measurements (six locations instead of four), they still assumed blubber was limited to the region between the neck and pelvis. They also measured and converted *TBW* to total fat mass based on an equation by Iverson et al. [26]: *Mass*_*fat*_ = *Mass*_*total*_− 1.37*TBW*, calculated from proportion water and fat-free tissue in gray seals (*Halichoerus grypus*) [27]. Comparisons between the two methods again produced similar results with error in the truncated cones method of 0.01% ± 4.25%, assuming the isotope dilution results were more accurate [21].

In general, while the truncated cones method has produced an accurate proxy for adult female adipose tissue and juvenile fat mass in elephant seals, the method cannot distinguish between different tissue types or provide a fully three-dimensional understanding of the animals. Namely, previous truncated cones methods for elephant seals have not accounted for skin and cannot differentiate between blubber and non-blubber adipose tissue. In addition, the truncated cones method has assumed that seals are circular when, in fact, gravity may play a role in creating a more elliptical shape when they are measured on land [28]. Differences in the assumption of shape could have a profound influence on estimates of volume and, thus, mass of different tissue types [29]. Lastly, previous methods for northern elephant seals used an ultrasound that did not produce an image [21, 24], making blubber depth measurements potentially less accurate and less precise. We aim to 1) determine if elephant seals are circular or elliptical while on land, 2) compare sculp depth measurements between an ultrasound that records a digital image and one that does not, providing a correction factor to account for differences, 3) measure the depth and fat content of elephant seal skin, 4) compare total proportion adipose tissue and proportion fat mass using three different techniques: a) the tritium-labeled water method, b) the traditional truncated cones method, and c) a modified cones method (Table 1), and 5) quantify which metrics create the most uncertainty in total proportion fat estimates for the modified cones method and the isotope dilution technique.

**Table 1:** Differences between traditional and modified truncated cones methods.

Parameter, Instrument, or Assumption	Traditional	Modified
Volume used	Neck to pelvis	Nose to tail
Shape	Circular	Elliptical
Ultrasound	No image	With image
Skin thickness	Not determined	1.32 ± 0.16 cm
Proportion fat in blubber	0.903	Early: 0.853 ± 0.026, Late: 0.823 ± 0.031
Blubber density	0.94 g·mL^-1^	0.89 ± 0.03 g·mL^-1^
Proportion fat in skin	Not determined	0.161 ± 0.007
Skin density	Not determined	1.17 ± 0.13 g·mL^-1^

## Methods

### Ethics statement

Animal handling protocols were approved by the University of California Santa Cruz Institutional Animal Care and Use Committee. The National Marine Fisheries Service authorized this research under the Marine Mammal Protection Act, permit 14636. To minimize stress, all procedures were performed under anesthesia [30].

During the molt haul out (April–June 2013), 14 adult females (9 early-molt and 5 late-molt) at Año Nuevo State Park, California, USA (37°7’59”N, 122°19’59”W) were immobilized with an intramuscular injection of 1.0 mg·kg^-1^ of tiletamine/zolazepam HCl. Sedation was maintained with subsequent intravenous injections of ketamine and diazepam (100 mg·mL^−1^ and 5 mg·mL^−1^) as needed [28, 31].

Total mass was measured using a carbon fiber tripod with a hand winch and a canvas sling attached to a calibrated tension dynamometer (MSI, Seattle, WA, USA; capacity 1000 ± 1 kg). Three seals’ mass measurements were taken in triplicate and indicated that repeated mass measurements varied less than the accuracy of the scale. Therefore, measurement uncertainty in total mass was not included in this analysis.

### Truncated cones

#### Data collection and sample processing

**Fig 1:**
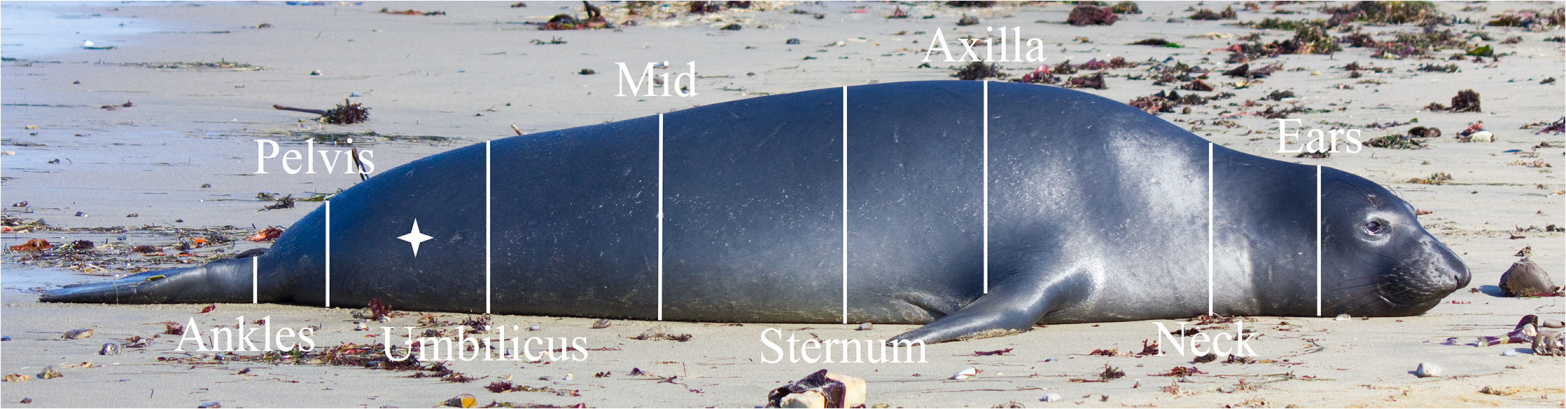
Morphometric measurement locations. Locations of width, height, girth, and curvilinear measurements. Dorsal and lateral ultrasound images were also taken at all locations except ears and ankles. Star indicates location of blubber biopsy. Photo by Patrick Robinson under NMFS Permit 14636.

Following Slip et al. [22] and Webb et al. [21], girth measurements were taken at eight predefined locations: ears, neck, axial, sternum, mid-seal, umbilicus, pelvis, and ankles (Fig 1 and [28]). Curvilinear length was measured from the tail to each girth location, and total body curvilinear length was measured to the tip of the nose. In order to determine circularity, eight height and width measurements were also taken at each girth location using two parallel poles on either side of the seal and one horizontal pole above the seal.

**Fig 2:**
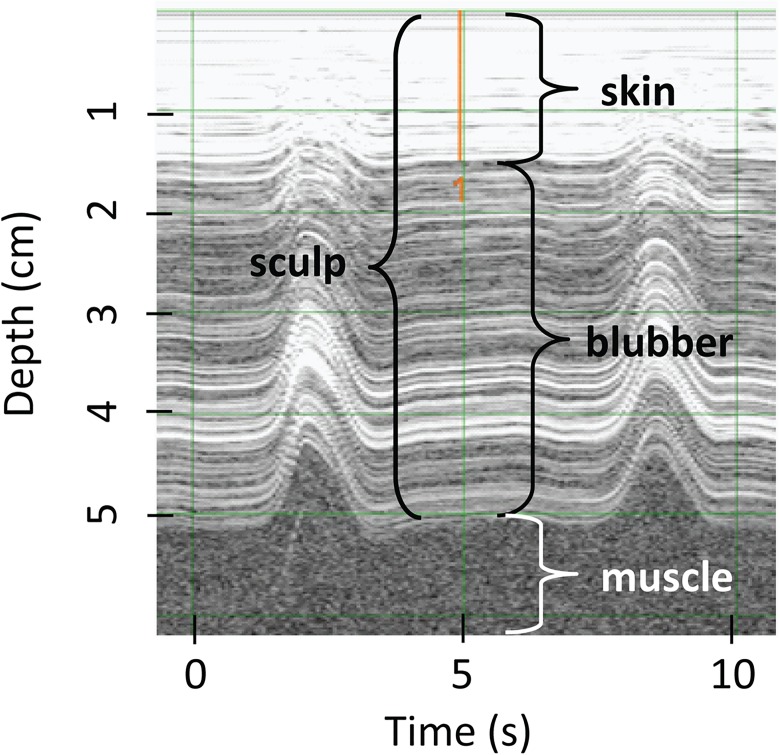
Ultrasound image. Lateral position at the sternum location along the length of the animal (see Fig 1), late-molt ultrasound image of blubber and skin layers. Peaks represent compressions.

To determine if blubber thickness changes over the body, ultrasound images were taken dorsally and on both sides of the seal at all predefined locations except ears and ankles using a Signos portable ultrasound that records a digital image of depth over time (Signostics Ltd., Colvelly Park, Australia). Thirteen of the 14 females were also scanned using a Scanoprobe II ultrasound (Scanco, Ithica, NY), which provides a reading of blubber depth via a line of red lights activated depending on the density of the tissue. The ultrasound technician determines the blubber depth based on changes in the on-off patterns of the lights, indicating a barrier between blubber and non-blubber tissue. All morphometric measurements, including ultrasound images, were independently collected three times by different researchers. Sculp thickness was later measured from ultrasound images using the SigViewer program (Fig 2; Signostics Ltd., Colvelly Park, Australia) independently by three different researchers.

Several other measurements were estimated from additional animals. Skin (epidermis and dermis) thickness was measured two independent times (late molt and early breeding seasons) from blubber core samples of 23 adult females. Skin thickness did not change during the post-molt foraging trip. Blubber density was determined from blubber samples taken from the lateral area between the pelvic and umbilical measurements of a fresh adult female carcass that had died from an apparent shark attack but was otherwise healthy. Blubber pieces (two with skin attached to measure sculp density, and four without skin) were weighed to the nearest 0.001 g, and volume was measured via water displacement in a 200 ml graduated cylinder accurate to 0.5 ml. Density of the skin was also estimated by taking five biopsy cores of carcass skin. Skin cores were weighed and volume was estimated based on the size of the biopsy punch and measured skin depth.

Proportion fat in blubber was measured from the same 46 blubber core samples used to estimate skin thickness. In calculations, we assumed proportion fat in blubber during the early molt was the same as during early breeding. Estimates of the proportion of fat in blubber by mass can vary widely between extraction techniques and even between studies using the same techniques [32]. We used the Soxhlet method to maintain consistency with studies that provide key data to translate total body water from labeled water techniques to amount of crude fat [33, 34] (S3 File). We used modified Folch extraction to estimate the proportion fat in the skin using three samples of skin from one carcass [35, 36] (S3 File). While different extraction techniques could result in different proportion lipid in skin, the overall contribution of skin to total body fat was low, so potential differences in extraction techniques would not greatly affect results.

Along with direct measures from carcass tissue, density of skin (*ρ*_*D*_) was also estimated using density of a core section of tissue including blubber and skin (*ρ*_*T*_), density of blubber only (*ρ*_*B*_), skin depth (*d*_*D*_), and depth of blubber and skin at the umbilicus (*d*_*T*_) (S4 File).

$$\rho_{D}=\rho_{T}+\left(\rho_{T}-\rho_{B}\right)\left(\frac{d_{T}-d_{D}}{d_{D}}\right)$$ (Eq 1)

#### Calculations: truncated cones

The truncated cones method involves several steps to estimate total proportion fat from sculp and blubber. First, morphometric measurements are used to calculate total body, skin, and blubber volumes. Then, skin and blubber mass are a product of volume and density of the respective tissues. Fat mass found in skin and blubber is a product of mass and the proportion fat in skin and blubber, respectively. Fat mass is then divided by total mass to get total proportion fat.

To estimate blubber volume from morphometric data, straight lengths between girth (or height-width) measurements (*L*_*S*_) were calculated using curvilinear length (*L*_*C*_) and the difference in radii between the two girth (or height) measurements (*r*), assuming the distances were short enough that curvature would not produce a measureable difference in results.

$$L_{s}=L_{C}^{2}-{\left(r_{1}-r_{2}\right)}^{2}$$ (Eq 2)

The total volume of each seal was calculated by adding up the volumes of each section of animal using both the elliptical and circular truncated cones methods [29]. Elliptical truncated cone volumes (*V*_*T*_) and end volumes based on an elliptical cone (*V*_*C*_) were calculated, where *a* is the radius on the long axis (seal width) and *b* is the radius on the short axis (seal height) of the ellipse.

$$V_{T}=\left(\frac{\pi \cdot L_{S}}{6}\right)\cdot \left(2a_{1}b_{1}+a_{2}b_{1}+b_{2}a_{1}+2a_{2}b_{2}\right)$$ (Eq 3)

$$V_{C}=\frac{1}{3}\left(\pi \cdot L_{S}\cdot ab\right)$$ (Eq 4)

For derivation, see S2 File. In the case of circular truncated cone volumes, *a*_1_ = *b*_1_ = *r*_1_, *a*_2_ = *b*_2_ = *r*_2_, and *a* = *b* = *r*.

Blubber volume was calculated in two different ways to compare traditional methods (circular cones, skin not accounted for, blubber only between neck and pelvis, no ultrasound image) with a modified method (elliptical cones, skin accounted for, blubber between ears and ankles, with ultrasound image) (Table 1). For both methods, an inner volume was calculated using the equations above but subtracting ultrasound sculp depth from the radii of girth or height-width measurements. The traditional method calculates volumes from the pelvis to the neck, while the modified method uses entire body volume estimates, assuming no blubber anterior to the ears and posterior to the ankles. The traditional method assumes the entire volume from the surface of the animal to the blubber-muscle boundary is blubber, so the blubber volume was the difference between total and inner volume. Only ultrasound measurements without digital images and circular cones were used in this first method. The modified method accounts for skin by creating a third volume where skin depth is subtracted from height-width measurements before estimating volume. Skin volume is the difference between the total volume and this new volume. Blubber volume is the difference between the new volume and the inner volume. This second method was done using the ultrasound with digital images.

To convert to mass of fat in blubber from blubber volume, blubber volume was multiplied by the density of blubber and the proportion fat in blubber. Traditional truncated cones fat mass was calculated as the outer circular cone volume including skin multiplied by the density of that volume from previous studies (0.94 g·mL^-1^) [23, 24] and assuming 90.3% ± 0.3 fat by mass [6, 21]. Blubber volume from the modified method was multiplied by pure blubber density newly measured from a carcass and newly measured proportion fat in blubber for early and late molt. Skin volume was also multiplied by skin density and proportion fat in skin to get fat mass in skin. Proportion fat was calculated as the fat mass divided by total mass (S1 Code).

### Tritiated water

#### Data collection and sample processing

After initial blood samples were taken from the extradural vein, 10 of the 14 seals received an IV injection of 2–14 ml of 0.8–1.2 mCi tritiated water prepared as sterile saline. The contents of the syringe were weighed prior to injection to the nearest 0.001 g, and the syringe was flushed several times to ensure that all the tritiated water was injected. Serial blood samples taken from two seals every 30 minutes for 3 hours showed no changes in tritiated water concentrations after 90 minutes (data not presented). After the 90 minute equilibration period for all other seals, blood samples (7–10 ml) were taken from the extradural vein and the hind flipper, both posterior to the injection site. Blood samples were collected without anti-clotting additive. While results from the extradural vein and hind flipper were equivalent, only results from hind flipper blood samples are reported here.

An additional 12 juvenile seals were dosed with tritiated water for an energetics study in May–March 2009 and 2010 [37]. Six seals were injected with 5.0 mCi tritiated water in 2009, and the remaining seals were given 1.0 mCi in 2010. Girths, curvilinear lengths, and Scanoprobe II ultrasound measurements without images were also taken at all predefined locations along the seals. This sample allowed us to retest the relationship between total proportion fat in sculp (cones method) vs. total proportion fat (tritiated water method) in juvenile elephant seals as described in previous studies [21].

Samples were kept on ice and processed immediately upon return from the field, 2–5 hours later. Blood was centrifuged for 10 min at 3000 rpm, and serum was transferred into plastic vials and then stored at -20° C. The serum was later thawed, and water within the serum distilled using the freeze-capture method of Ortiz et al. [16] (S5 File). Specific activity for each sample (counts per million, or *CPM*) was measured using a Rack Beta Spectral-Liquid Scintillation Counter. Water samples from the same serum or standard were run in triplicate with the scintillation counter programmed to estimate *CPM* over a 30 minute period three times for each sample. Counting vials were kept at room temperature in closed boxes or within the dark Scintillation Counter until processed.

#### Calculations: tritiated water

To estimate proportion fat from tritiated water values, *TBW* (in ml) is a function of the specific activity injected (*CPM*_*inj*_), the specific activity concentration after equilibrium (*CPM·ml* ^*-1*^_*eq*_), and background specific activity concentration levels (*CPM·ml* ^*-1*^_*bg*_) (S2 Code):

$$TBW=\frac{CPM_{inj}}{CPM\cdot ml_{eq}^{-1}-CPM\cdot ml_{bg}^{-1}}$$ (Eq 5)

*CPM*_*inj*_ is calculated first by estimating the specific activity of the tritiated water stock (*CPM·ml* ^*-1*^_*stock*_) using standards (S2 Code). *CPM·ml* ^*-1*^_*stock*_ was given a normal distribution, calculating the mean and standard deviation using standard replicates.

To remove the potential for negative estimates of proportion fat and to quantify uncertainty, we reanalyzed original published data comparing proportion water estimated from tritium dilution ($P_{H_{2}O}$) and the logit of proportion fat (P_*fat*_).

$$\text{ln}\left(\frac{P_{fat}}{1.0-P_{fat}}\right)=\alpha_{0}+\alpha_{1}\left(\frac{P_{H_{2}O}}{1.0-P_{H_{2}O}}\right)+\left(\frac{P_{H_{2}O}}{1.0-P_{H_{2}O}}\right)\varepsilon$$ (Eq 6)

Proportion fat was determined by extracting crude fat (Soxhlet technique) from homogenized whole pinniped carcasses [33, 34, 38]. We estimated the uncertainty in the relationship between the log of the fat ratio and the water ratio calculated from tritiated water using data from weaned or adult gray seals (n = 2; [33]), Antarctic fur seals (*Arctocephalus gazella*, n = 5; [34]), and ringed seals (*Pusa hispida*, n = 2; [38]). Based on results from other species, error was assumed to expand at higher water ratios (L. Schwarz, unpublished analysis). Measurement uncertainty was not available for this portion of the analysis.

Results were compared with the Iverson et al. [26] equation and an equation based on guinea pig data from Pace and Rathbun [19] that has also been used in elephant seal research [16, 24, 39]. The equation from guinea pig data determines proportion adipose tissue and proportion fat from proportion water in adipose tissue, proportion water in non-adipose tissue, and proportion fat in adipose tissue, all available as beta distributions with uncertainty. Therefore, we could also determine uncertainty in the Pace and Rathbun equation and compare it with the uncertainty from our analysis (S1 File and S3 Code). The same parameters (with and without uncertainty) from Pace and Rathbun [19] were used to convert proportion water to proportion adipose tissue for our elephant seal sample. When estimating proportion adipose tissue, proportion water estimates from tritiated water were first adjusted by 0.967 to account for bias compared to proportion water measurements from homogenized carcasses [40].

### Additional analyses

Variability in final estimates of total proportion fat mass and proportion adipose tissue mass was calculated using Monte Carlo simulation with parameters sampled from a normal distribution, or beta distribution for proportions, with means and variances calculated from the data (N = 15000). In the case of tritiated water results and ultrasound image readings, there was a hierarchical relationship in error (three independent estimates from three separate measurements for each animal). However, hierarchical analysis was not possible for these estimates due to small sample size. Instead, all nine estimates were treated as independent, and mean and variance of the normal, or lognormal for ultrasound readings, distribution were calculated from all nine estimates. To determine the sensitivity of total proportion fat to uncertainty in all measurements, each measurement for each animal was allowed to vary for 15,000 iterations based on quantified uncertainty while all other values were held constant at their mean values.

A simple linear regression *Y* = *β*_0_ + *β*_1_ *X* + *ε* statistically described the relationship between blubber volume using the traditional truncated cones method (*X*) and the modified cones method (*Y*). To compare sculp depth measurements between the ultrasound device without an image (*X*_*U*_) and with an image (*Y*_*U*_), an additional linear regression equation was analyzed with the assumption that the slope of the relationship (*β*_*U*_) was constant across all body locations (*i*): *Y*_*U*,*i*_ = *β*_*U*_ *X*_*U*,*i*_ + *β*_*i*_ + *ε*. The Bayesian models assumed a normal distribution on error with uniform priors on all parameters and a uniform prior greater than zero on the inverse of the variance. Posterior distributions and joint posterior samples of parameters were determined using the program MTG (Metropolis within Gibbs) developed by D. Goodman of Montana State University [41]. Standard practices (multiple independent chains with low lag-1 autocorrelation) ensured mixing, convergence, and stationarity in posterior samples [42–44].

## Results

### Truncated cones

**Fig 3:**
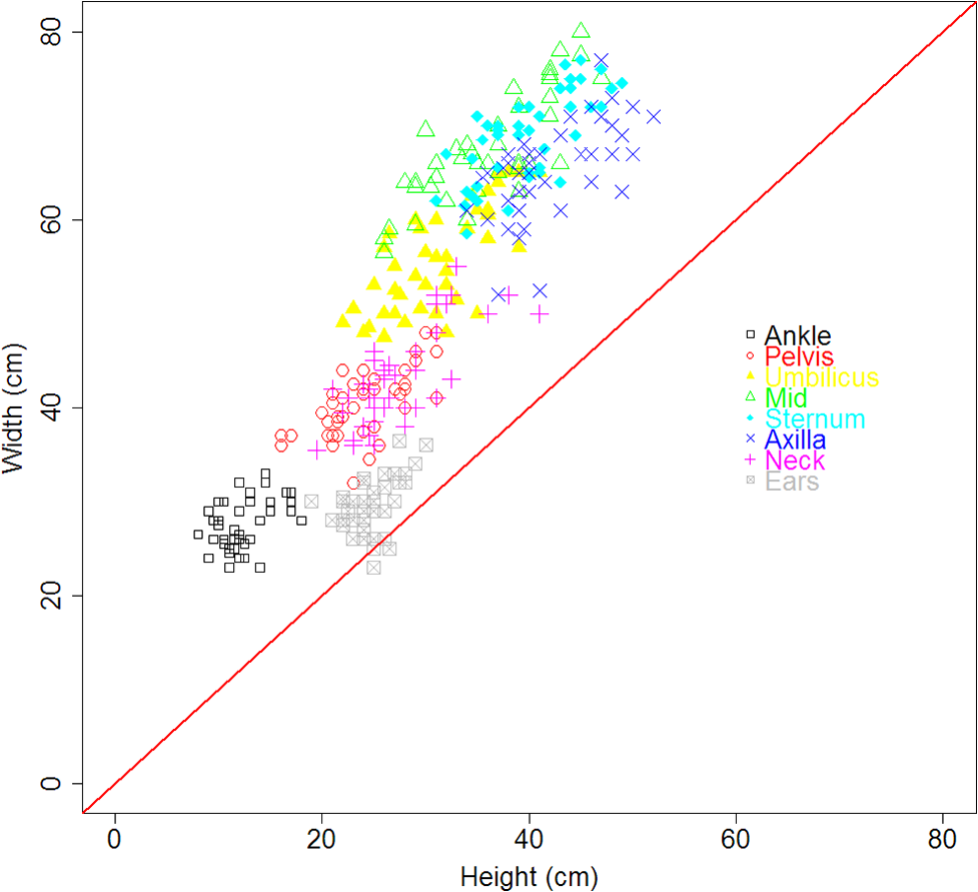
Measures of circularity. Width vs. height at different locations along an elephant seal’s body (*N* = 14 animals, each animal independently measured three times). Points along the diagonal red line indicate circularity.

Morphometric data showed that adult female northern elephant seals are not circular when hauled out on land, with the potential exception of the head (‘Ears’ in Fig 3). Dividing measured total seal mass by total elliptical truncated cone volume produced a mean total body density of 1.11 ± 0.07 g·mL^−1^ at the beginning of the molt and 1.19 ± 0.06 g·mL^−1^ at the end of the molt. When the traditional circular truncated cone volume estimates were used for the entire body, this resulted in a mean total body density of 0.88 ± 0.03 with no difference between early and late molt. Standard deviations in total body density were slightly higher using elliptical cones (0.03 ± 0.01) compared to circular cones (0.02 ± 0.01).

**Fig 4:**
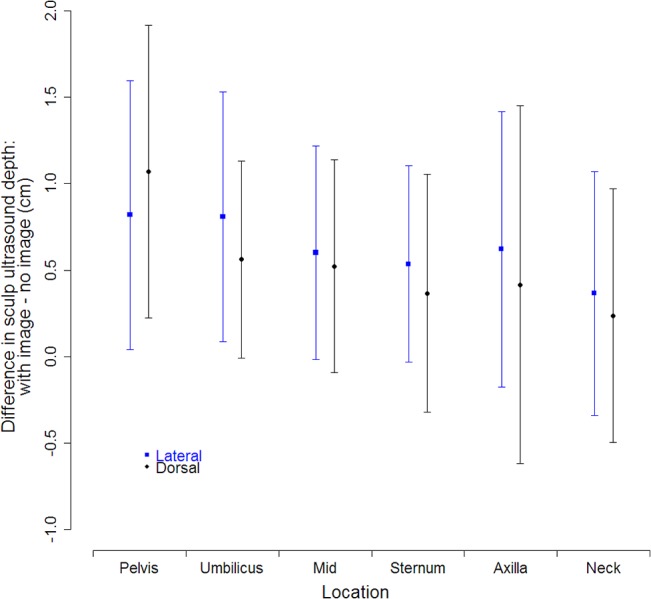
Difference in sculp ultrasound thickness comparing ultrasounds with and without an image for dorsal and lateral surfaces of the seals from pelvis to neck. Points are means, and bars represent ± 1 standard deviation combining distributions of triplicate measures for dorsal surfaces and six measures for lateral surfaces from 13 seals.

Skin thickness was 1.32 ± 0.16 cm (*N* = 46). Sculp thickness was greater using the ultrasound with a digital image compared to an ultrasound without an image (Fig 4). Hierarchical Bayesian linear regression analysis that accounts for uncertainty in measurements suggested a larger difference in ultrasound reading for smaller depths in the lateral view while differences in dorsal view readings were more consistent (Table 2).

**Table 2:** Posterior marginal distributions of parameters relating sculp depth ultrasound measurements from images (*Y*_*U*,*i*_) to ultrasound measurements without images (*X*_*U*,*i*_) at different locations (*i*): pelvis, umbilicus, mid, sternum, axilla, and neck, on the dorsal and lateral surfaces.

Surface	Parameter	Marginal	Marginal standard	Parameter correlation						
		mean	deviation	*β*_*pelvis*_	*β*_*umbilicus*_	*β*_*mid*_	*β*_*sternum*_	*β*_*axilla*_	*β*_*neck*_	$\sigma_{\varepsilon }^{2}$
Dorsal	*β*_*U*_	8.73E-01	8.71E-02	-0.866	-0.949	-0.978	-0.961	-0.917	-0.975	-0.079
	*β*_*pelvis*_	1.26E+00	2.89E-01		0.822	0.853	0.839	0.797	0.848	0.098
	*β*_*umbilicus*_	9.33E-01	3.22E-01			0.928	0.915	0.866	0.924	0.090
	*β*_*mid*_	8.51E-01	3.37E-01				0.939	0.899	0.953	0.077
	*β*_*sternum*_	8.41E-01	3.60E-01					0.882	0.938	0.086
	*β*_*axilla*_	8.27E-01	3.58E-01						0.894	0.070
	*β*_*neck*_	5.40E-01	3.66E-01							0.140
	$\sigma_{\varepsilon }^{2}$	2.12E-03	5.79E-03							
Lateral	*β*_*U*_	5.83E-01	9.46E-02	-0.889	-0.927	-0.947	-0.959	-0.933	-0.939	-0.017
	*β*_*pelvis*_	1.95E+00	3.08E-01		0.827	0.843	0.859	0.826	0.838	0.012
	*β*_*umbilicus*_	2.13E+00	3.65E-01			0.875	0.892	0.866	0.866	0.037
	*β*_*mid*_	2.15E+00	3.86E-01				0.908	0.888	0.888	0.025
	*β*_*sternum*_	2.21E+00	3.81E-01					0.901	0.904	0.017
	*β*_*axilla*_	2.23E+00	3.76E-01						0.880	0.019
	*β*_*neck*_	2.00E+00	3.92E-01							0.020
	$\sigma_{\varepsilon }^{2}$	1.24E-03	4.47E-03							

*Y*_*U*,*i*_ = *β*_*U*_ *X*_*U*,*i*_ + *β*_*i*_ + *ε* where $\varepsilon ∼N\left(0,\sigma_{\varepsilon }^{2}\right)$. Measurement uncertainty was included since locations were measured in triplicate.

**Fig 5:**
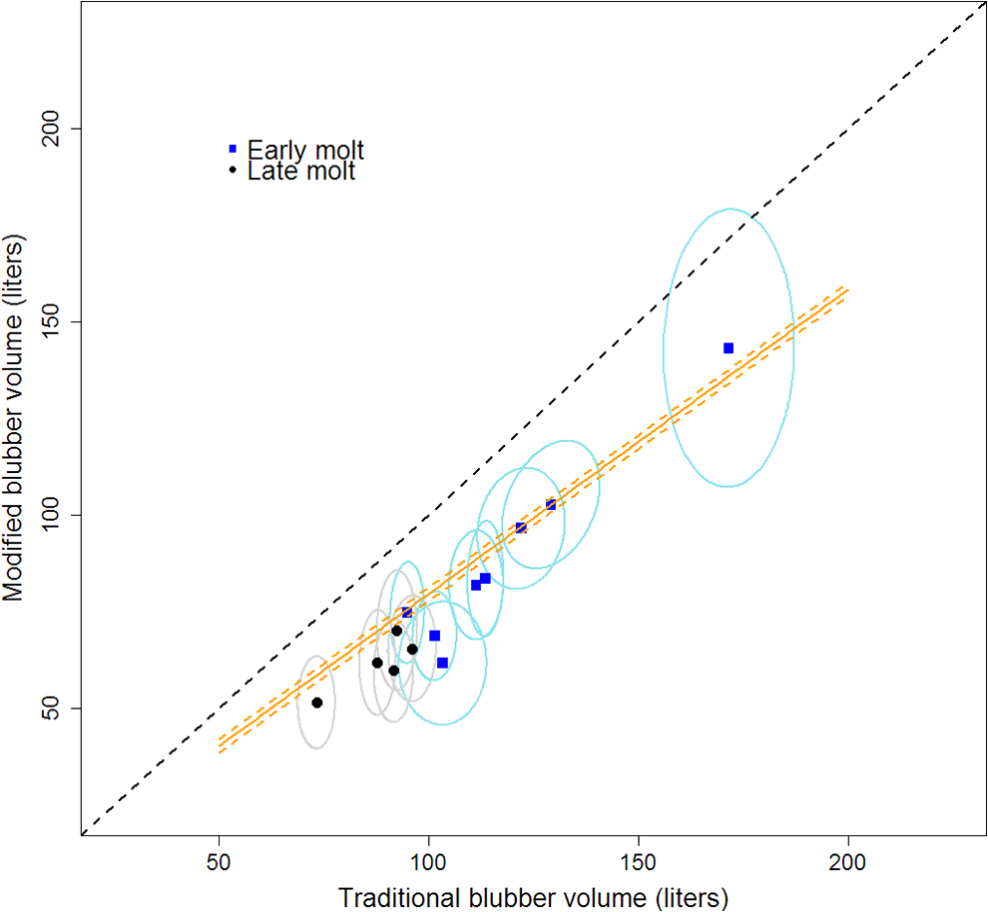
Comparison of truncated cones techniques to estimate blubber volume. Traditional truncated cones technique (circular cones without ultrasound images, not accounting for skin, blubber from neck to pelvis) vs. a new method (elliptical cones with ultrasound images, accounting for skin, blubber from ears to ankles). Points are means and circles are 95% posterior intervals. Dashed black line is parity. Orange lines are mean (solid) and 95% posterior interval (dashed) values from a hierarchical Bayesian analysis accounting for uncertainty in measurements.

The traditional cones method defines blubber as the volume between the pelvis and neck and from the surface of the skin to the muscle, whereas the modified cones method defines blubber as the volume from ankles to ears and from the skin-blubber interface to the muscle. In addition, body shape and type of ultrasound were different between the two methods. Combining all differences, the traditional truncated cones method produced higher blubber volume estimates compared to the modified cones method (Fig 5). The marginal posterior distributions for parameters were 0.90 ± 0.10 for slope (*β*_*1*_) and -18.1 ± 10.8 for intercept (*β*_*0*_), and parameters were highly negatively correlated (-0.98). Posterior variance distribution was 1.2 ± 5.7, leading to a well-defined relationship between the two methods (Fig 5). The modified cones method determined that 7.0 ± 1.4% of blubber volume is outside the neck to pelvis region and also estimates that skin makes up 11.7 ± 1.6% of total volume before molt and 13.6 ± 1.7% after molt.

For the single fresh carcass, blubber density was 0.89 ± 0.03 g·mL^-1^, and sculp density was 0.93 ± 0.00 g·mL^-1^. Skin density was 1.17 ± 0.13 g·mL^-1^, and proportion fat in skin was 0.161 ± 0.007. Skin density based on the properties of a blubber and skin core (Eq 6) was 1.02 ± 0.08 g·mL^-1^. Late-molt adult females had a proportion fat in blubber of 0.823 ± 0.031 (*N* = 23), while early-breeding females had a slightly higher proportion fat in blubber of 0.853 ± 0.026 (*N* = 23).

Using total volumes, densities, and proportion fat in different tissue types, sculp has a mean of 57.8 ± 4.6% fat in early molt and 52.2 ± 4.1% fat in late molt. Sculp density is 0.98 ± 0.05 g·mL^−1^ during early molt and 1.00 ± 0.06 g·mL^−1^ during late molt. However, both sculp percent fat and density will potentially vary across the seal with changes in blubber and skin thickness. Based on measured total mass and the modified cones method, the density of non-blubber tissue (including skin) is 1.18 ± 0.10 g·mL^−1^ during early molt and 1.28 ± 0.09 g·mL^−1^ during late molt.

### Tritiated water: water to fat

**Fig 6:**
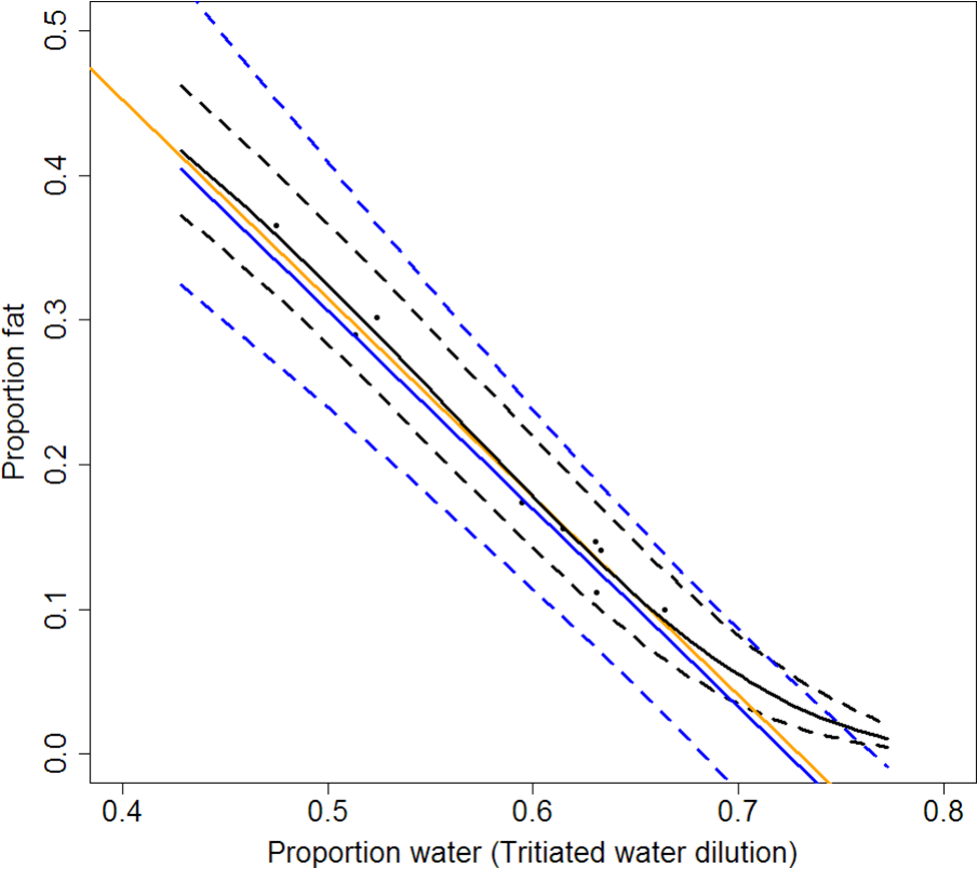
Proportion fat vs. proportion total body water. Relationship between proportion total body water as measured from tritiated water dilution and proportion fat from dissection. Points are data for three pinniped species. Solid black line is mean and dashed lines are 95% posterior intervals from results of Eq 6. The orange line represents the estimated linear relationship without uncertainty by Iverson et al. [26]: Mass_Fat_ = Mass_Total_− 1.37Mass_Water_. The solid blue line is the estimated mean value of proportion fat given proportion water from guinea pig carcasses [19] with 95% posterior intervals (dashed blue lines). For posterior distributions of Eq 6 parameters, see Results. For the guinea pig equation derivation and R code, see S1 File and S3 Code.

The mean estimate of proportion fat vs. proportion water using a logit function and water ratio was similar to estimates from a linear relationship without uncertainty developed by Iverson et al. [26] for grey seals (Fig 6). In addition, our results closely matched the function for guinea pigs based on water content of different tissues and proportion fat in adipose tissue (Fig 6). Our estimates diverged from the other estimates at higher proportion water values (> 0.66) where the logit function does not allow for negative values of proportion fat. The marginal posterior distributions for parameters defined in Eq 6 were 0.86 ± 0.14 for slope (*α*_*1*_) and -1.6 ± 0.16 for intercept (*α*_*0*_), and parameters were highly negatively correlated (-0.97). Posterior uncertainty is relatively high but declines with higher values of proportion water as proportion fat asymptotes to zero (from ± 0.04 95% PI at $P_{H_{2}O}$ = 0.5 to ± 0.01 at $P_{H_{2}O}$ = 0.8). Uncertainty in proportion fat is higher using the function derived from guinea pig estimates of water content and proportion fat.

### Method comparisons: proportion adipose tissue and proportion fat

**Fig 7:**
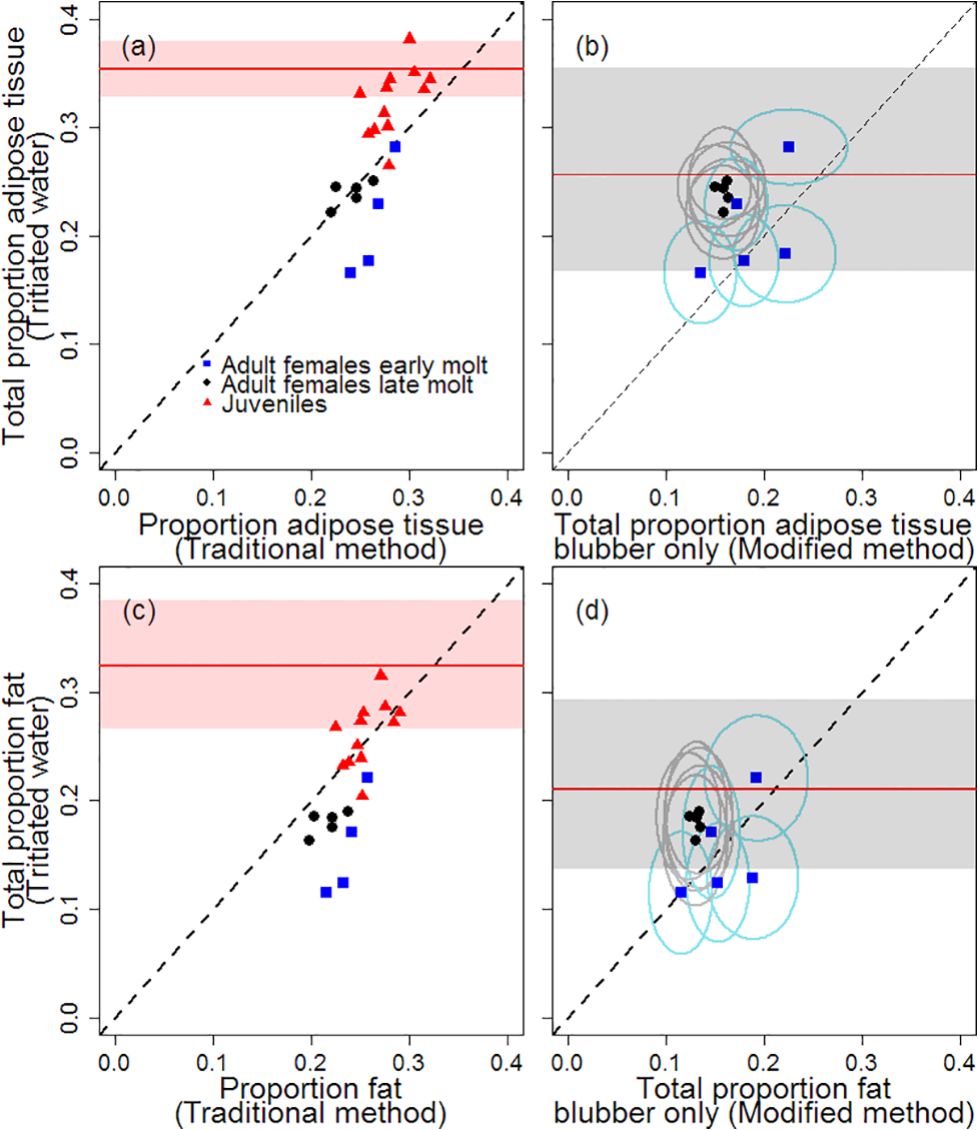
Comparison of techniques to measure proportion adipose tissue and proportion fat. Proportion adipose tissue (top) and proportion fat (bottom), showing results from the traditional truncated cones method (left) and the modified cones method (right) compared to labeled water estimates. Points are means, and circles represent 95% posterior range. Dashed diagonal line indicates a 1:1 relationship. Red shaded area with red line indicate mean and 95% confidence interval for juveniles reported by Webb et al. [21] using the tritiated water method. Gray shaded area with red line indicate mean and 95% posterior interval for molting adult females reported by Worthy et al. [24] using the tritiated water method.

For both proportion adipose tissue and proportion fat, estimates from both cones methods produced slightly higher mean values for females during early molt compared to late molt (Fig 7). Tritiated water and traditional truncated cones estimates of proportion adipose tissue were consistent for late-molt females only, while estimates for early-molt females were lower for tritiated water. Estimates of juvenile proportion adipose tissue were generally higher using tritiated water compared to traditional truncated cones methods (Fig 7A). Juvenile proportion fat estimates were comparable between tritiated water and traditional truncated cones methods, while the traditional cones method consistently produced higher adult female proportion fat (Fig 7C).

As long as adipose tissue and fat exist outside the blubber layer, the modified cones method should produce a lower estimate of proportion adipose tissue and fat (since it measures only blubber) compared to the tritiated water method which measures fat for the entire body. However, early molt females do not fit the expected pattern, with the modified cones method producing values at or over the tritiated water method values. Calculating the ratio of blubber values (modified cones method) to total estimates (tritiated water method) and combining posterior distributions for late molt females, adipose tissue consists of 66.6 ± 9.1% blubber by mass, and 74.2 ± 14.4% of total body fat is found in blubber (Fig 7B and 7D). Based on skin density, skin volume, and proportion fat in skin, 12.2 ± 2.9% of fat for late molt females is found in the skin. In general, proportion fat uncertainty is higher for the labeled water technique (standard deviation: 0.024 ± 0.002) compared to the modified cones technique (standard deviation: 0.015 ± 0.003).

### Sensitivity

**Fig 8:**
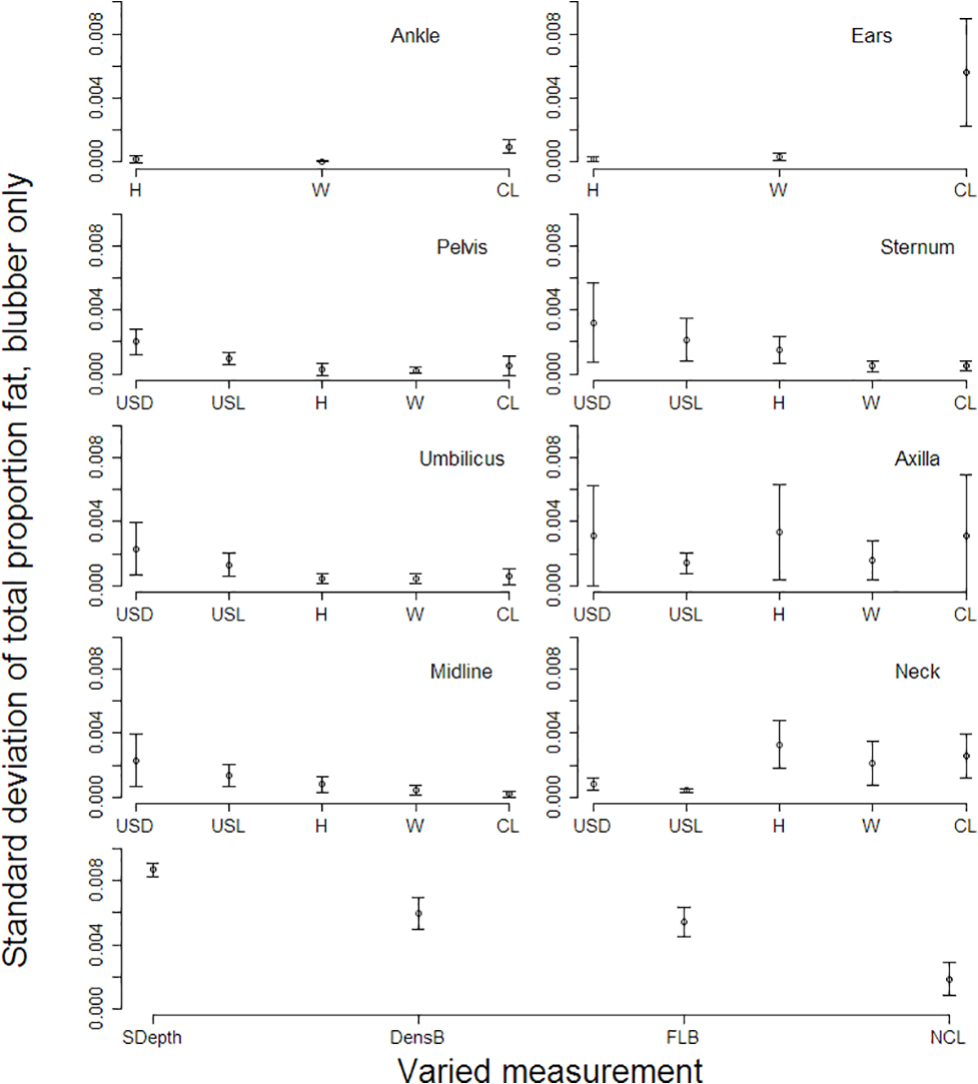
Truncated cones sensitivity. Standard deviation of total proportion fat when measurements are independently varied using the modified truncated cones method. Height (H), width (W), dorsal ultrasound measurement (USD), and lateral ultrasound measurement (USL) refer to elliptical truncated cone measurements. CL = curvilinear length segment, SDepth = skin depth, DensB = density of blubber, FLB = proportion fat in blubber, NCL = curvilinear length from ears to tip of nose. Points are means and bars are standard deviations for all measured animals (*N* = 14).

**Fig 9:**
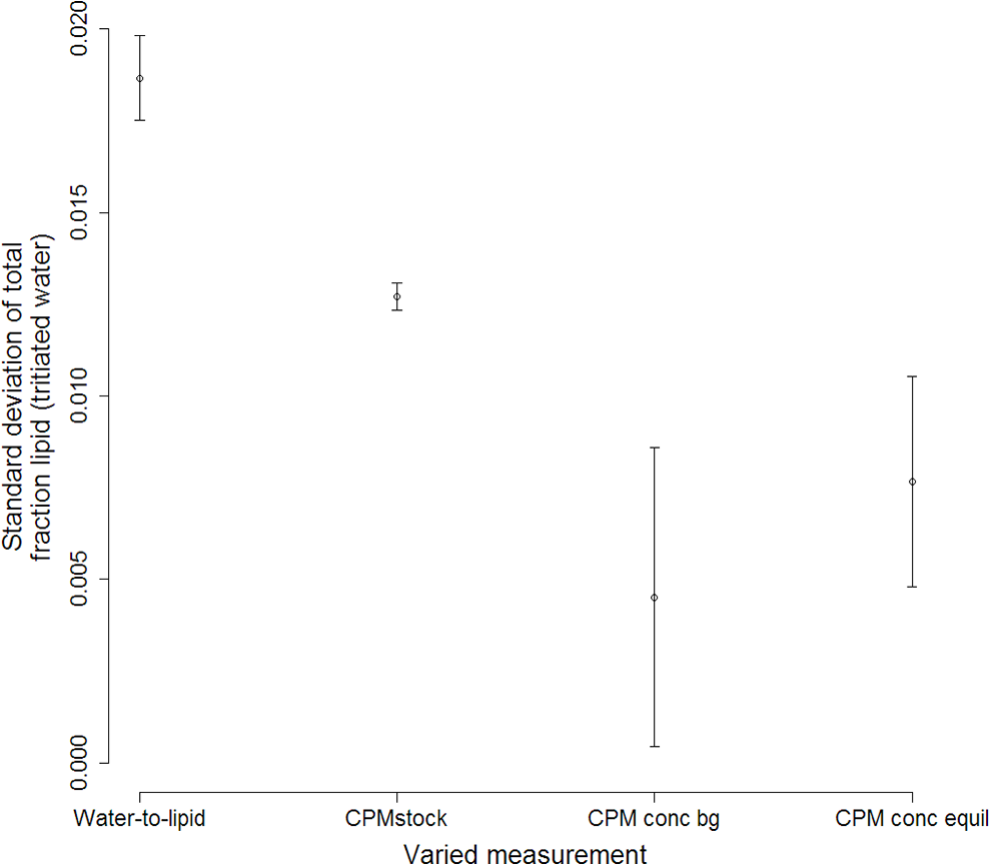
Tritiated water sensitivity. Standard deviation of total proportion fat from tritiated water method when measurements are independently varied. Water-to-fat is the uncertainty associated with the relationship between proportion water and proportion fat, as measured in Eq 6 with results in Fig 6. CPMstock is derived from standard solutions. CPM conc bg is the background CPM·mL^−1^, and CPM conc equil is the CPM·mL^−1^ after the labeled water had equilibrated in the animal. Points are means and bars are standard deviations for all measured animals (*N* = 10).

While variability in any one measurement did not create a high degree of variability in total proportion fat estimates from the modified cones method, estimates were most sensitive to uncertainty in skin depth, density of blubber, and proportion fat in blubber (Fig 8). Also, as curvilinear length reaches longer distances (tail-to-axilla, tail-to-neck, and tail-to-ears), measurement variability increases, leading to a higher sensitivity to such measures for overall proportion fat estimates. Higher variability in some other measurements (ultrasound dorsal measurements at the sternum and axilla, and height at the axilla and neck) also contribute to higher uncertainty in proportion fat estimates. Uncertainty in the water-to-fat function (Eq 5 and Fig 6) created the most variability in total proportion fat estimates using the labeled-water method, followed by variability in the specific activity of the tritiated water injectate standard solutions (Fig 9).

## Discussion

When estimating blubber volume, there are four differences between the traditional truncated cones method and the modified cones method: body shape (circular vs. elliptical), ultrasound type (without vs. with image), volume used (neck to pelvis vs. nose to tail), and skin (included as part of blubber vs. considered a separate tissue) (Table 1). Additionally, blubber density and proportion fat in blubber (used to estimate blubber mass and fat mass) are different between the two methods. The logit function converts water mass to adipose tissue mass or fat mass while preventing negative mass values. Lastly, these new methods quantify measurement and process uncertainty for all steps and parameters.

Comparing elliptical and circular truncated cones, our results followed the same pattern observed by Shero et al. [28] for Weddell seals (*Leptonychotes weddellii*): seals are not circular when hauled out on land, and volume estimates were lower using elliptical compared to circular cones. That difference leads to higher estimates of total body density using elliptical cone volumes when compared to estimates using circular cone volumes. Elephant seals become less dense (more buoyant) as they accumulate fat over their foraging trips [21, 45, 46]. Based on drift rates from dives during the foraging trip, Crocker et al. [45] found that animals remain negatively buoyant during the short post-breeding trip, while they become neutrally buoyant near the end of the longer post-molt trip [45, 46]. Overall our elliptical total body density estimates were high compared to the density of seawater, which is consistent with animals remaining negatively buoyant during the post-breeding trip. In contrast, total body density estimates from circular cones suggested that animals were positively buoyant, near the density of blubber.

If seal body density decreases in response to successful foraging and resulting fat accumulation, it follows that total body density will increase while seals are fasting, as indicated by our results using elliptical volumes. In contrast, body density using circular cones did not change over the fast. These data suggest that elliptical cone volume estimates are a more accurate representation of body composition than circular cone volumes. We therefore recommend the use of elliptical cone volumes when estimating mass and body composition of elephant seals in particular and more likely phocid seals in general.

Ultrasound sculp measurements in northern elephant seals were thicker using an ultrasound that produces a digital image compared to an ultrasound that does not (Table 2, Fig 4). If sculp depth is the primary metric of interest, we have provided a function with appropriate uncertainty to convert non-image measurements to with-image values. When placing the ultrasound scanner perpendicular to the muscle and using a high gain, ultrasound images have the ability to produce a clean boundary between blubber and muscle (Fig 2). In addition, an archived image provides a permanent record of the measurement that can be reviewed and discussed by multiple technicians. The ultrasound without an image may have been sensitive to connective tissue within the blubber layer, leading to shallower sculp depths. Overall, we recommend the use of imaging ultrasound technology with archival capabilities.

The thickness of the skin layer is notable in elephant seals. For example, our data suggest that in the lateral region between the pelvic and umbilical measurements (where blubber cores are typically collected), skin may be between 30 and 37% of the depth of an ultrasound measurement. However, that percentage would change with differences in blubber and skin thickness across the body, leading to changes in sculp density and proportion fat across the body. Therefore, it would be difficult, if not impossible, to estimate the contribution of sculp to adipose tissue and fat mass without explicitly accounting for skin. For example, Shero et al. [28] used the modified cones method when estimating sculp volume (did not measure or account for skin depth) in Weddell seals. Their total proportion fat found in sculp varied depending on the mass of the animal, with smaller animals having smaller proportions. This may be due to the relatively higher proportion of skin the smaller animals have, rather than a reduction in the proportion fat available as energy.

Since skin contributes variably to ultrasound depth measurements but does not contain much fat, we suggest treating it as a separate tissue from blubber. In addition, further studies should determine if skin thickness varies across an elephant seal, as seen for Steller sea lion pups (*Eumetopias jubatus*) [47]. Advances in portable ultrasound devices can allow us to identify different tissues, particularly skin, which may allow us to account for such variability in the future [48].

Two additional skin properties are important for measuring the contribution of sculp, rather than only blubber, to mass and proportion lipid values. Prior to this study, proportion fat in skin had not been reported for elephant seals, but our estimates were the same as those reported for grey seals (0.162 ± 0.074) [33]. Our measures of skin density (1.17 ± 0.13 g·mL^-1^) are higher than that of Worthy et al. [24], who reported elephant seal skin as positively buoyant (0.87 g·mL^−1^ with an assumed skin depth of one cm), which more closely matches what we found for blubber density (0.89 ± 0.03 g·mL^-1^). Our total density for the combination of skin and blubber from a carcass sample (0.93 g·mL^−1^) is similar to that reported by Worthy et al. [24] for northern elephant seals (0.94 g·mL^−1^) and Gales and Burton [23] for southern elephant seals (0.95 g·mL^−1^). It is unclear if their estimates included the skin, but given the differences in our density estimates for skin and blubber, it is likely they included skin. We also calculated sculp density using total tissue volumes from elliptical truncated cones combined with specific tissue densities. The density of sculp using whole body measurements is slightly higher but with high uncertainty (early molt: 0.98 ± 0.05 g·mL^−1^, late molt: 1.00 ± 0.06 g·mL^−1^) compared to the carcass tissue measurements. The estimated density using total body measurements (which accounts for differences in blubber layer thickness throughout the body) may be more appropriate when considering the entire body. In addition, when estimating the contribution of sculp to total lipid mass, we recommend using the new estimates of percent fat in sculp reported here (early molt: 57.8 ± 4.6%, late molt: 52.2 ± 4.1%), rather than assuming the sculp mass has the same proportion fat as blubber (early molt: 82.3 ± 3.1%, late molt: 85.3 ± 2.6%).

While proportion fat in blubber can be highly variable even between studies of the same species, our values are similar to measurements taken in healthy mature female Weddell seals, ringed seals, early-lactating gray seals, and weaned elephant seal pups [28, 49–51], and our measurements fall within the range of values generally seen for phocids [32]. The traditional cones method used values from previous studies that measured the content of water in blubber and assumed the remaining mass was fat [6, 21]. Our study specifically isolates fat from blubber and has the largest sample size with the highest precision to date for adult female elephant seals. As seen in odontocetes and phocids (including this study), the proportion fat in blubber may vary depending on age, sex, reproductive status, fasting condition, time of year, and health [28, 49, 52]. Therefore, direct blubber core samples for all individuals for which fat mass will be estimated from truncated cones would be the ideal way to obtain this metric. If it cannot be measured directly, estimates should come from a representative sample. Even within this study, we assumed early molt was equivalent to early breeding proportion fat in blubber, which may not be the case.

Combining all differences (body shape, ultrasound type, volume used, and skin) in blubber volume calculations, the traditional truncated cones method produced higher blubber volume estimates compared to the modified cones method (Fig 5). Along with higher estimated blubber volumes, the traditional method used higher blubber density values and higher proportion lipid in blubber compared to the modified method (Table 1), leading to higher total proportion adipose tissue and total proportion fat estimates (Fig 7). Our results confirm that the traditional cones method provides proportion adipose tissue estimates equivalent to that measured by tritiated water methods for late-molt females (Fig 7A). In addition, the traditional cones and tritiated water methods produce similar total proportion fat estimates for juveniles (Fig 7C), although our percent error (7.6 ± 7.1%) is higher than previously reported [21]. However, the traditional cones method does not work in all cases. For example, it does not provide accurate estimates of proportion adipose tissue for juveniles or proportion fat for late molt females, nor does it provide an accurate representation of the amount of adipose tissue or fat in blubber, skin, or sculp. Lastly, the idiosyncrasies of the traditional method may mean the technique will not perform well on many phocid species. Overall, the traditional technique may provide an accurate proxy for two elephant seal metrics, but it has its limitations.

Early-molt female results were not consistent with tritiated water results with both cone methods estimating a higher proportion adipose tissue and proportion fat. The cone methods are consistent with each other (Fig 5), suggesting that there was a bias or error in the tritiated water method. We could not isolate any source of error, as the difference between the measurements was not a function of the tritiated water stock used, the amount or concentration of injected labeled water, the size of the animal, or the order or date in which the animals were sampled or the samples were processed. However, as we did not know how long the females had been on the beach, they may not have been in a completely fasting state, and water in the gut would have led to an underestimate of total adipose tissue and fat. Water in the gut leads to a higher estimate of total body water which results in an underestimate of proportion fat. In addition, early molt animals may be in a better hydration state than fasting animals. In such a case, the equations relating water to adipose tissue or fat may be different, and the current equation would lead to an underestimate of adipose tissue and fat.

Using the tritiated water method, Webb et al. [21] reported generally higher overall percent adipose tissue estimates for juveniles compared to this study (35.4 ± 1.3% vs. 28.5 ± 2.2%) (Fig 7A and 7C). Similar to Webb et al. [21], we tended to see higher overall percent adipose tissue estimates in larger juveniles. Percent adipose tissue declines over the fast, and Worthy et al. [21] targeted large animals at the beginning of the fast. In the current study, juvenile seals were sampled from early- through mid-fast and tended to be smaller than those sampled in the Webb et al. [16] study (175.6 ± 28.9 kg vs. 186.4 ± 32.7 kg). In addition, pup mass and percent lipid can vary between years, and lipid metabolism varies with pup size [53]. However, we could find no statistical explanation for the difference in adipose tissue estimates between our study and those of Webb et al. [16], including a mass, age, or timing effect. Even so, our values fall within the range seen for the entire time period [21].

As with juvenile seals, the tritiated water method yielded estimates of total proportion fat for late molt females (mean: 0.180 ± 0.011) within the lower range of previously published values for adult female and male elephant seals [24, 39, 54]. Worthy et al. [24] found a total proportion fat of 0.21 ± 0.04 (Fig 7B and 7D) for adult female elephant seals during the molt. Using proportion water from tritiated water dilution reported in Costa et al. [39] and the results of Eq 6, we estimated total proportion fat for late lactating females (0.21 ± 0.07). Likewise, Crocker et al. [54] measured a slightly higher proportion fat for adult male elephant seals at the end of the breeding fast (0.204 ± 0.007) compared to our estimates. The three studies did not quantify uncertainty, and given the small sample sizes and high amount of uncertainty in our estimates, the small differences are difficult to compare.

A primary objective of this study was to present a modified truncated cones method and calculations that can be used for a variety of species because they provide a more comprehensive understanding of morphology and energy storage. Our modified cones method isolates blubber and skin volume and uses blubber density and proportion fat measurements to determine the role of blubber and skin in total fat content. Using the modified cones technique, the percentage of total fat found in skin (12.2 ± 2.9%) and blubber (74.2 ± 14.4%) for late molt females is consistent with what has been reported for healthy gray seal carcasses (skin: 10.2 ± 7.7%, blubber: 74.4 ± 7.4%, *N* = 8) [33]. Using the traditional cones method, previous studies state both species lack fat stores outside the hypodermis [24, 55]. However, the total percentage fat found in blubber reported here and from gray seal carcasses is actually lower than that calculated for fasting harp seal (*Pagophilus groenlandicus*) pup carcasses (90.1 ± 2.6%), a phocid species known to rely heavily on non-blubber lipid stores during fasting [56]. Our results indicate around 14% of fat may be stored outside the hypodermis in elephant seals. Overall, we recommend using the modified technique, particularly because it should be transferable to a variety of species and will be useful when blubber and the properties of blubber are of interest, such as in toxicological studies.

We found a very strong relationship between the traditional and modified methods that could allow us to transfer between the techniques. In cases where width and height measurements are not available (only girth measurements), we recommend calculating “blubber” volume using the traditional method and then utilizing the relationship between traditional blubber volume and blubber volume calculated with the modified method (Fig 5 and Results). The modified blubber volume is then multiplied by new estimates of blubber density and proportion fat in blubber.

### Uncertainty

Another objective of this study was to quantify the uncertainty in measurements often used to represent energy reserves, which are important metrics in some conservation models, and determine if some of that uncertainty could be reduced. Posterior uncertainty was high for both the modified cones and labeled-water techniques. For example, the higher 95% posterior proportion fat value for late molt females is 2.5 times larger than the lower 95% posterior proportion fat value in the case of labeled-water results, which has important implications for understanding energetic requirements and translating those requirements to survival and reproduction.

Sensitivity analysis gives us the ability to determine which parameter variabilities lead to the greatest variance in the outcome. It also provides us with a way to determine if new procedures or methods to measure the input variables can dramatically reduce the uncertainty in estimates of proportion fat. In this particular system, three of the modified truncated cones parameters creating the most uncertainty in total proportion fat are not measured for each individual: skin depth, density of blubber, and proportion fat in blubber (Fig 8). Likewise, variability in tritiated water results is more sensitive to parameters not measured directly from the animal: the water-to-fat function (Eq 5, Fig 6) and the determination of the specific activity of the tritiated water injectate (Fig 9). Reduced uncertainty in those parameter estimates could reduce uncertainty in proportion fat results.

Measuring some of the above metrics for each individual could reduce uncertainty. Skin depth could be measured from skin plug samples collected during blubber biopsies. In the event that blubber biopsies are not routinely collected, skin depth measurements for individuals could be estimated from ultrasound images, and ultrasound image results could be compared with measures of skin plug samples or from measures of skin depth from a small incision [48]. The challenges of obtaining estimates of blubber density are associated with the need for a large volume of tissue, which often cannot be safely collected from living animals. In addition, the tissue is no longer viable for many other purposes once it has been submerged in water, so determining density estimates for live individuals may not be feasible. Current sample sizes for density estimates are minimal: one carcass each in this study and Gales and Burton [23], and an unknown sample size for Worthy et al. [24]. Therefore, more measurements of this kind would be useful. Sensitivity to longer curvilinear lengths was due to high variability in those measurements for specific animals. The result calls attention to the need to measure those lengths more carefully in the future.

Along with quantifying measurement uncertainty in the labeled-water method, we calculated and incorporated the process uncertainty in the relationship between total body water and total body fat. The level of posterior uncertainty in the proportion fat vs. proportion water logit function was relatively high. Our small sample size contributes somewhat to the high variability. However, studies of other mammalian species with larger sample sizes indicate that variability in this relationship may be even higher and is particularly high at higher proportion water values [57]. There are no studies that measure uncertainty in the proportion total body water to proportion fat relationship which, if accounted for in analyses, could reduce uncertainty in the process. The logit function itself also plays a role in the high level of posterior uncertainty. A linear function would have lower posterior variability, but allows negative proportion fat results at higher proportion water values. In this case, multiplicative vs. non-multiplicative error was not a contributing factor in the posterior uncertainty since samples did not have very low proportion fat values. Future work could investigate the possibility of improving the model fit by switching between the logit and linear functions at the point at which the lower 95% limit of the functions intersect. Such results would reduce uncertainty in proportion fat (or total body fat) estimates calculated from labeled water techniques while disallowing negative values. However, our function has less uncertainty compared to the relationship based on proportion water in different tissues in guinea pigs that has been used extensively.

Another source of error is in the determination of the specific activity of the injectate. The injectate is too concentrated to count directly in a scintillation counter, so the determination of its specific activity required dilution to levels equivalent to the initial specific activity measured in the animals’ body fluids. I.e. we prepared standard solutions to roughly mimic the concentrations of tritiated water we expected to see in the seal (0.01 ml of injectate to 850–1200 ml DI water). Small errors in the preparation of the standard dilution can introduce larger errors into the calculation of TBW. Thus, each injectate standard should be measured multiple times with as much accuracy and precision as possible. Incomplete blood distillation can introduce a low bias in background and equilibrium activity, leading to an overestimate of total body water which produces an underestimate of adipose tissue and fat. Multiple distillation samples and testing the distillation process with known quantities of labeled water can determine if incomplete distillation is occurring (S5 File). Lastly, the scintillation counter itself produces variability which can be reduced with longer count periods and higher specific activity doses. In addition, such variability can be quantified with multiple samples measured multiple times, as we have done.

Uncertainty in body condition metrics has larger implications in our understanding of how species interact with and adapt to a variable environment that could also include human-caused disturbance. In our case, the amount of energy potentially needed to successfully reproduce has a range that could be 2.5 times greater than the lowest estimate. Females that have to accumulate more fat to reproduce have lower reproductive resilience during times of poor foraging and are more sensitive to disturbances that reduce foraging. In addition, a population that requires more energy will affect its environment differently compared to populations that consume less energy. Lastly, along with our understanding of physiological processes and pathways, these body condition metrics can be an integral part of species and ecosystem management models because they provide a measure of individual fitness that can translate to a population.

## Supporting Information

S1 File Proportion water to proportion fat. (DOCX)

S2 File Calculating the volume of an elliptical truncated cone. (DOCX)

S3 File Fat extraction methods. (DOCX)

S4 File Biopsy core to skin density. (DOCX)

S5 File Water distillation and standards processing. (DOCX)

S1 Code Truncated cones.R. (R)

S2 Code Tritiated water to TBW.R. (R)

S3 Code Pace&Rathbun.R. (R)

S1 Data Animal_Inj_Data. (TXT)

S2 Data Blood_CPM. (TXT)

S3 Data MorphData. (TXT)

S4 Data Standard_CPM. (TXT)

S5 Data StandardDilution. (TXT)

S6 Data UltrasoundImageData. (TXT)
