# Supplementary material for: Comparisons and Uncertainty in Fat and Adipose Tissue Estimation Techniques: The Northern Elephant Seal as a Case Study
Source: PLoS One. 2015 Jun 29;10(6):e0131877. doi: 10.1371/journal.pone.0131877 (PMC4486730; doi:10.1371/journal.pone.0131877)
Supplement: S1 File — (DOCX) [file pone.0131877.s001.docx]

**S1. Calculating proportion fat from proportion water in different body cavities**

Given

*TBW* = total body water mass

*M_T_* = total mass

*PW_T_* = proportion water in total body mass = *TBW*/*M_T_*

*M_A_* = mass of adipose tissue

*PW_A_* = proportion water in adipose tissue by mass

*M_NA_* = mass of non-adipose tissue = *M_T_* - *M_A_*

*PW_NA_* = proportion water in non-adipose tissue by mass

*PF_A_* = proportion fat in adipose tissue by mass

*M_F_* = mass of fat

*PF_T_* = proportion fat in total body mass = *M_L_*/*M_T_*

$$TBW={PW}_{A}\cdot M_{A}+{PW}_{NA}\cdot M_{NA}$$

Replace *M_NA_*.

$$TBW={{PW}_{A}M}_{A}+{PW}_{NA}\left( M_{T}-M_{A} \right)$$

Solve for *M_A_*.

$$M_{A}=\left( \frac{{PW}_{NA}M_{T}}{{PW}_{NA}-{PW}_{A}} \right)-\left( \frac{TBW}{{PW}_{NA}-{PW}_{A}} \right)$$

Solve for *M_F_* and divide both sides by *M_T_*.

$${M_{F}={PF}_{A}M}_{A}={PF}_{A}\left[ \left( \frac{{PW}_{NA}M_{T}}{{PW}_{NA}-{PW}_{A}} \right)-\left( \frac{TBW}{{PW}_{NA}-{PW}_{A}} \right) \right]$$

$${PF}_{T}=\left( \frac{{PW}_{NA}{PF}_{A}}{{PW}_{NA}-{PW}_{A}} \right)-\left( \frac{{PF}_{A}}{{PW}_{NA}-{PW}_{A}} \right)\cdot{PW}_{T}$$

Pace and Rathbun ([1945](#_ENREF_1)) report means and standard deviations for all parameters for guinea pigs. Reported values were translated in to beta distributions to represent uncertainty. See R code for values.

Pace, N. and E. N. Rathbun. 1945. Studies on body composition 3. The body water and chemically combined nitrogen content in relation to fat content. Journal of Biological Chemistry 158:685-691.
