# Supplementary material for: Comparisons and Uncertainty in Fat and Adipose Tissue Estimation Techniques: The Northern Elephant Seal as a Case Study
Source: PLoS One. 2015 Jun 29;10(6):e0131877. doi: 10.1371/journal.pone.0131877 (PMC4486730; doi:10.1371/journal.pone.0131877)
Supplement: S2 File — (DOCX) [file pone.0131877.s002.docx]

**S2. Calculating the volume of an elliptical truncated cone**

b

B

a

h

A

$$V=\int_{0}^{h} \pi xydz$$

*x* and *y* change linearly as a function of *z*.

$$y=m_{b}z+B$$

$$x=m_{a}z+A$$

At z= h, $b=m_{b}h+B$ and $a=m_{a}h+A$. By rearrangement, $m_{b}=\frac{b-B}{h}$ and $m_{a}=\frac{a-A}{h}$.

Make substitutes for x and y:

$$V=\int_{0}^{h} \pi\left[ \left( \frac{a-A}{h} \right)z+A \right]\left[ \left( \frac{b-B}{h} \right)z+B \right]dz$$

By integration:

$$V=\left. \frac{\pi}{h^{2}}\left\{ \frac{z^{3}}{3}\left( a-A \right)\left( b-B \right)+\frac{z^{2}h}{2}\left[ A\left( b-B \right)+B\left( a-A \right) \right]+h^{2}ABz \right\} \right]_{z=0}^{z=h}$$

Which becomes

$$V=\frac{\pi h}{6}\left( 2ab+Ab+Ba+2AB \right)$$
