# Supplementary material for: Comparisons and Uncertainty in Fat and Adipose Tissue Estimation Techniques: The Northern Elephant Seal as a Case Study
Source: PLoS One. 2015 Jun 29;10(6):e0131877. doi: 10.1371/journal.pone.0131877 (PMC4486730; doi:10.1371/journal.pone.0131877)
Supplement: S3 File — (DOCX) [file pone.0131877.s003.docx]

**S6. Fat extraction methods**

*Soxhlet*

Blubber core samples frozen at -20° C for one to two years were thawed and processed in Belgium in conjunction with a toxicological study. Since blubber cores were only weighed after freezing, we tested potential mass loss due to the freezing process. Blubber core samples weighed before and after five weeks of freezing (*N* = 10) showed no mass loss due to freezing.

Blubber cores were weighed without the skin (*M_core_*) and desiccated by homogenization with Na_2_SO_4_. Mixtures were then placed in Whatman thimbles and spiked with internal standards. Soxhlet extraction using hexane:acetone (3:1 volume ratio) took two hours, isolating lipid from the Na_2_SO_4_ and non-lipid tissue. The resulting extract was transferred to clean, pre-weighed test tubes, rinsing Soxhlet containers twice with hexane to limit loss of extract. Solvents were concentrated using warm N_2_ gas, and the extract was weighed (*M_extract_*). An aliquot of extract was then separated and weighed (*M_aliquotwet_*). The aliquot was evaporated at room temperature until visually dry, was further dried at 110°C for one hour, and reweighed (*M_aliquotdry_*). The four mass measurements were used to determine proportion lipid in the blubber (see Calculations section).

*Folch*

We used the Folch technique to estimate the proportion lipid in the skin using three samples of skin from one carcass. Pre-weighed skin samples (0.5 g), with the stratum corneum removed, were mashed in 10.5 ml of 2:1 chloroform:methanol. Then 2.6 ml of 0.7% NaCl_aq_ was added, and the solution was vortexed for 5 – 10 s. After centrifuging for 20 min at half speed, most of the upper aqueous, polar phase (containing no lipids) was removed using a glass pipette. The non-polar lower phase was removed using a new glass pipette while not disturbing the interface. The lower phase was filtered through anhydrous sodium sulfate using a funnel and filter paper (Whatman), rinsing ten times with chloroform to ensure lipid was not left in the filtration system. The solvent was captured in a pre-weighed kimax test tube and evaporated under nitrogen in a water bath at 25 – 30°C. The test tube was reweighed, and the lipid mass was calculated as the change in mass.
