# Supplementary material for: Comparisons and Uncertainty in Fat and Adipose Tissue Estimation Techniques: The Northern Elephant Seal as a Case Study
Source: PLoS One. 2015 Jun 29;10(6):e0131877. doi: 10.1371/journal.pone.0131877 (PMC4486730; doi:10.1371/journal.pone.0131877)
Supplement: S4 File — (DOCX) [file pone.0131877.s004.docx]

**S7. Calculating density of the skin layer from a biopsy core**

Given

*ρ_T_* = density of a core of blubber and skin (known)

*ρ_B_* = density of blubber (known)

*ρ_D_* = density of skin

*m_D_* = mass of skin layer

*m_B_* = mass of blubber layer

*V_D_* = volume of skin layer

*V_B_* = volume of blubber layer

*d_D_* = depth of skin layer (measured)

*d_T_* = depth of skin and blubber layer combined (measured)

$$\rho_{T}=\frac{m_{D}+m_{B}}{V_{D}+V_{B}}$$

By rearrangement

$$m_{D}=\rho_{T}\left( V_{D}+V_{B} \right)-m_{B}$$

By definition

$$m_{B}=\rho_{B}\cdot V_{B}$$

By substitution and dividing by skin volume

$$\rho_{D}=\frac{m_{D}}{V_{D}}=\rho_{T}+\left( \rho_{T}-\rho_{B} \right)\left( \frac{V_{B}}{V_{D}} \right)$$

For any core, $V=\pi\cdot r^{2}\cdot d$, where r is the radius of the biopsy punch, and by substitution

$$\rho_{D}=\rho_{T}+\left( \rho_{T}-\rho_{B} \right)\left( \frac{d_{T}-d_{D}}{d_{D}} \right)$$
