# Supplementary material for: Comparisons and Uncertainty in Fat and Adipose Tissue Estimation Techniques: The Northern Elephant Seal as a Case Study
Source: PLoS One. 2015 Jun 29;10(6):e0131877. doi: 10.1371/journal.pone.0131877 (PMC4486730; doi:10.1371/journal.pone.0131877)
Supplement: S5 File — (DOCX) [file pone.0131877.s005.docx]

**S8. Water distillation and standards processing**

Water was extracted from serum samples of 420 μL. While the amount of resulting distillate could vary between serum samples, the quantity of sample sent for analysis was standardized to 300 μL in 20 mL counting vials to remove any potential variability caused by differences in sample volume. Two animals were dosed with a lower specific activity of tritiated water and required higher volumes (500 μL) of distillate to achieve comparable CPMs. Therefore distilled samples were combined to achieve three replicated 500 μL for analysis.

Incomplete evaporation can cause isotope fractionation [[20](#_ENREF_20)] causing lower HTO values. Therefore, to determine if sample was lost during distillation, all materials were weighed before and after the evaporation process. Sample serum loss was 2.2 % ± 2.5 SD, and there was no correlation between the amounts of sample lost and labeled water values. To ensure all water had been distilled, remaining samples from two animals were distilled a second time. In addition, the remaining samples after the second distillation were placed in a warming oven at 94° C for 30 minutes. No measurable amount of water was left in the Teflon plugs after the first distillation. Lastly, three serum samples with a known quantity of tritiated water were distilled and compared with tritiated water concentrations from pure water samples with the same amount of tritiated water. Results were identical, indicating no or minimal fractionation.

Two stocks of HTO were used for this study: three animals were dosed with one stock, and the remaining seven animals were dosed with the second stock. The specific activity of each HTO stock was measured by diluting the injectate in to standard samples created based on the masses of the animals and the amount of tritiated water injected.

Given

*m_inj_* = mass of injected tritiated water (g)

*m_stock_* = pre-determined mass of tritiated water stock solution (= 0.01 g)

*m_animal_* = mass of the animal (g)

*C_stock_* = unknown concentration of tritiated water stock

Assuming an animal is roughly 66% water, the concentration of tritiated water in the seal (*C_seal_*) is calculated as

$$C_{seal}=\frac{C_{stock}\cdot m_{inj}}{0.66{\cdot m}_{animal}}$$

For a standard, we emulate the concentration of tritiated water in the seal. We need to determine the amount of deionized water that replicates the seal (*m_DI_*), given we will add a known amount of tritiated water stock (*m_stock_*).

$$C_{seal}=\frac{C_{stock}\cdot m_{stock}}{\left( m_{DI}+m_{stock} \right)}$$

By substitution,

$$m_{DI}=\frac{0.66\cdot m_{animal}\cdot m_{stock}}{m_{inj}}-m_{stock}$$

Either 300 or 500 μL, depending on the animal’s dosage, of each standard were added to counting vials in triplicate. Two standards were mixed for each animal mass and tritiated water stock (n = 4 standards for stock 1, for a total of 36 replicates; n = 5 standards for stock 2, for a total of 45 replicates).

Fifteen ml of scintillation cocktail (EcoLite+) were added to each counting vial and the mixture was blended well. Tests with standardized serum distillate showed that the ratio of distillate to cocktail did not affect results. *CPM*s were measured using a Rack Beta Spectral-Liquid Scintillation Counter. Water samples from the same serum or standard were run in triplicate with the scintillation counter programmed to estimate *CPM* over a 30 minute period three times for each sample. Counting vials were kept at room temperature in closed boxes or within the dark Scintillation Counter until processed.
